# Preoperative Anxiety and Postoperative Pain in Patients With Laparoscopic Hysterectomy

**DOI:** 10.3389/fpsyg.2021.727250

**Published:** 2021-10-11

**Authors:** Lin Zhang, Li-Jun Hao, Xiao-Lai Hou, Ya-Ling Wu, Lu-Shi Jing, Ruo-Nan Sun

**Affiliations:** ^1^Department of Anesthesiology, Shanxi Provincial People’s Hospital, Taiyuan, China; ^2^Department of Pain, Shanxi Provincial People’s Hospital, Taiyuan, China; ^3^Department of Gynecology, Shanxi Provincial People’s Hospital, Taiyuan, China; ^4^Department of Psychology, Chengdu Medical College, Chengdu, China

**Keywords:** laparoscopes, preoperative anxiety, postoperative pain, hysterectomy, dexmedetomidine

## Abstract

**Objective:** This study was designed to investigate preoperative anxiety situations and postoperative pain degree in Chinese patients undergoing laparoscopic hysterectomy and to analyze the related factors of preoperative anxiety and the correlation between preoperative anxiety and postoperative pain to provide a reference for effective postoperative analgesia management.

**Methods:** A total of 100 female patients undergoing laparoscopic hysterectomy were enrolled in this study and randomly divided into two groups (*n* = 50, each). In group A, the patients were treated with dexmedetomidine and sufentanil for postoperative analgesia. In group B, the patients were treated with sufentanil alone for postoperative analgesia. All patients were evaluated with a self-rating anxiety scale (SAS) 1 day before the operation. The patients’ pain was evaluated using the numerical rating scale (NRS) 1 day after the operation, and data were recorded.

**Results:** In these 100 patients, the highest preoperative SAS score was 48, and the average score was 40.99 ± 4.55 points, which is higher than the norm in China. There were significant differences in preoperative SAS scores among patients with different occupations and previous surgical experience (*P* < 0.05). There was no significant difference in SAS scores among patients with different education levels (*P* > 0.05). The postoperative NRS score of group A was significantly higher than that of group B, and the difference was statistically significant (*P* < 0.05). The correlation coefficients between SAS scores and NRS scores in groups A and B were 0.836 and 0.870, respectively, presenting with a significantly positive correlation.

**Conclusion:** Preoperative anxiety is an important predictor of postoperative pain. Patients undergoing laparoscopic hysterectomy have preoperative anxiety. The degree of anxiety is influenced by the occupation and previous operation experience of the patients, and patients with higher preoperative anxiety have greater postoperative pain. In addition, we should not neglect the management of postoperative pain because of the small trauma of laparoscopic surgery, and dexmedetomidine combined with sufentanil can improve the postoperative analgesic effect.

## Introduction

Anxiety as a disorder is defined as a neurosis characterized by anxious overconcern extending to panic and frequently associated with somatic symptoms (Zung, 1971). Anxiety disorder is considered a common mental disease (Pan et al., 2019). According to the China Mental Health Survey (Huang et al., 2019), the prevalence of anxiety disorder in Chinese patients is 7.6%. As a strong stressor, surgery will inevitably cause anxiety. Previous studies revealed that the incidence of preoperative anxiety in patients with an elective surgery is high, and female patients are more prone to anxiety (Gan et al., 2014; Mimic et al., 2018). As early as the 1980s, studies revealed that the more anxious the emotion was, the lower the pain threshold of the body (Scott et al., 1983; Johnston, 1986; Browne, 2013). Most subsequent studies have reached the same conclusion (Caumo et al., 2002; Sobol-Kwapinska et al., 2016). Other studies have revealed that for different types of surgery, preoperative anxiety has different impacts on postoperative pain (Pinto et al., 2015). Therefore, we started this study to explore the correlation between the two further.

Previous studies on the correlation between anxiety and pain were mostly based on open surgery (Kain et al., 2000; Pinto et al., 2015). In recent years, with the development of minimally invasive technology, laparoscopic surgery has become the main surgical method. Therefore, this study focused on the impact of preoperative anxiety on postoperative pain in Chinese patients undergoing laparoscopic surgery. The investigators analyzed the preoperative anxiety of patients with different education levels, occupations, and surgical experiences. This can help us more accurately identify anxiety-prone people. Due to the short pain time after laparoscopic surgery (Hartwig et al., 2017), our study was limited to 24 h after surgery and did not extend to a longer time. The effects of two different postoperative analgesia schemes were also compared to provide a reference for clinical effective postoperative analgesia management.

## Materials and Methods

### General Information

The ethics committee of Shanxi Provincial People’s Hospital approved the present study. A total of 100 female patients who underwent elective laparoscopic hysterectomy from 2019 to 2020 in our hospital were enrolled in this study. The inclusion criteria were ASA grade I-II, age 45–55 years, and a weight of 50–80 kg. The exclusion criteria were patients with chronic pain, malignant tumors, and severe complications. These patients were randomly divided into two groups: dexmedetomidine postoperative analgesia pump group (group A) and sufentanil postoperative analgesia pump group (group B), with 50 patients in each group. Eligible patients were screened 1 day before surgery and informed of the investigation protocol in written and oral form. After signing the consent form, the patients filled in the questionnaire form about their medical history, socio-economic status, and SAS (Zung, 1971). For the patients who had difficulty filling in the form, researchers read it out and helped fill it in according to the patients’ answers. The returned incomplete questionnaire forms were timely supplemented on site. One day after the operation, the pain was assessed with the NRS (Suffeda et al., 2016; Gravani et al., 2020; Shen et al., 2020) when the patient was awake.

### Anesthesia Protocol

After the patients entered the operating room, routine electrocardiographic monitoring was performed. All patients were given total intravenous anesthesia. Propofol was used for anesthesia induction. Atracurium *cis-*benzene sulfonate and sufentanil were used for injection. Anesthesia was maintained with continuous infusion of propofol and remifentanil, and atracurium *cis-*benzene sulfonate and sufentanil were intermittently added on time and on-demand. Anesthesia was stopped at the end of the operation. The formula of postoperative analgesia pumping in group A was 1 μg/kg of dexmedetomidine, 1.2 μg/kg of sufentanil, and 10 mg of azasetron. The formula of postoperative analgesia pumping in group B was 1.2 μg/kg of sufentanil and 10 mg of azasetron. All the anesthetics were diluted with normal saline until the total volume was 100 mL. The loading dose was 2 mL, the background dose was 2 mL/h, the bolus dose was 2 mL/time, the lockout time was 15 min, and the limit dose was 10 mL/h.

### Data Acquisition

#### General Information Record Form of Operated Patients

General information included medical insurance status, marital status, education level, occupation, and previous surgery history.

#### Assessment of Preoperative Psychological Status

The SAS was developed by Zung (1971). It includes 20 items, 15 of which are positive scores and five of which are negative scores. Each item is scored to four levels. The rules are as follows: a score of 4 points means anxiety all or most of the time, a score of 3 points indicates that the patient has anxiety for a considerable time, a score of 2 points means anxiety for a short time, and a score of 1 point means no anxiety or anxiety for a very short time. The SAS score is calculated as follows: the scores of 20 questions are added, the sum is multiplied by 1.25, and the result is rounded. The final total is the SAS score.

#### Evaluation of Postoperative Pain

Numerical rating scale is a widely used pain assessment method worldwide. It has the characteristics of high reliability and sensitivity. On this scale, a scale of 0–10 is used to mark the pain intensity of different degrees, which the patient identifies. A score of 0 represents no pain, and 10 represents the most severe pain. A score below 4 indicates mild pain (pain does not affect sleep). A score of 4–7 indicates moderate pain, and a score of >7 indicates severe pain (pain leads to an inability to sleep or waking from sleep).

#### Statistical Analysis

The sample size was determined based on our preliminary data. The statistical analysis was performed using the SPSS 25.0 software. The “Summary *T*-Test” in SPSS was used to compare the average values. All data were expressed as mean ± SD. The statistical significance was *P* < 0.05. A *t*-test was used to compare the anxiety status of the subjects and the Chinese norm and compare the analgesic effects of the two analgesic schemes. One-way analysis of variance was used to find the independent factors related to preoperative anxiety. Pearson’s correlation analysis was used to explore the correlation between preoperative anxiety and postoperative pain.

## Results

### Sample Characteristics

Some demographic characteristics are also associated with a higher incidence of mental illness (Hah et al., 2020). Table 1 summarizes the demographic characteristics of the patients. In addition, in this study, potential confounding variables that may lead to postoperative pain were controlled, such as age, weight, ASA grade, and smoking history (Caumo et al., 2002; Kalkman et al., 2003; Ip et al., 2009; Yang et al., 2019; Table 1).

**TABLE 1:** Descriptions of general demographic features of the study sample.

	Variable	*n* (%)
Age (years)	45–50	39 (39%)
	51–55	61 (61%)
Weight (kg)	50–65	52 (52%)
	66–80	48 (48%)
Smoking	Yes	10 (10%)
	No	90 (90%)
Marital status	Yes	95 (95%)
	No	5 (5%)
Health insurance status	Yes	100 (100%)
	No	0 (0%)
ASA status	I	10 (10%)
	II	90 (90%)

### Preoperative Psychological Characteristics

The results of the SAS of 1,158 average Chinese patients measured by the Scale Collaboration Group were used as the norms in China (Zhang, 1998). In this study, the SAS score of 100 patients undergoing laparoscopic hysterectomy was 40.99 ± 55 points, compared with the norm (29.78 ± 46) in China. Preoperative anxiety of patients was higher than that of the norm, and the difference was statistically significant (*P* < 0.01, Table 2).

**TABLE 2:** Sample’s level of preoperative anxiety and depression.

	*N*	SAS	*n* (%)	Mean ± SD	*t*-value	*p*-value
Surgical patients	100	≤ 30 31–40 41–49 ≥50	0% 41% 59% 0%	40.99 ± 4.55	24.626	0.000*
The norm	1185			29.78 ± 0.46		

SAS, the self-rating anxiety scale; SD, standard deviation. * represents *P* < 0.01, and the difference is statistically significant.

**FIGURE 1:**
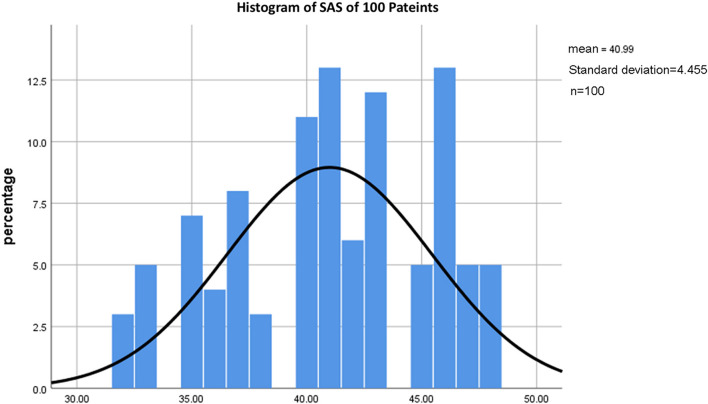
Histogram of SAS of 100 patients. The histograms of the SAS scores of all patients showed that all patients had a score less than 50. The highest preoperative SAS score was 48. Patients with a SAS score of 48 accounted for 5% of all cases.

In addition, the results of the present study revealed that for these 100 patients, the SAS score was 32–48. No patients had SAS scores lower than 30, and no patients had SAS scores higher than 50 (Figure 1).

### Univariate Analysis of Preoperative Anxiety

The influence of education level (three categories), occupation (four categories), and previous operation experience (three categories) on preoperative anxiety were analyzed. The result revealed that unemployed patients had higher preoperative anxiety. The lowest SAS score in this category was 40. Patients with previous painful surgery experience had the highest degree of anxiety. The lowest SAS score in this category was 45. The patient’s occupation and previous surgical experience affected their preoperative anxiety, and the difference was statistically significant (*P* < 0.01). The education level of patients had no impact on preoperative anxiety (Table 3).

**TABLE 3:** Univariate analysis of preoperative anxiety-related factors.

	Variable	*n*	SAS	Lowest value	Highest value	*p*-value
Education level	Primary school and below	33	41.12 ± 4.23	32	48	0.399
	Junior high school and senior high school	35	40.20 ± 4.65	33	47	
	Junior college or above	32	41.66 ± 4.41	32	48	
Occupation	Enterprises and institutions	18	36.39 ± 4.17	32	46	0.000*
	Worker	32	39.25 ± 3.63	32	46	
	Peasant	30	43.77 ± 2.88	37	48	
	Unemployed person	20	43.80 ± 2.71	40	48	
Operation experience	No history of surgery	38	36.24 ± 2.50	32	40	0.000*
	With a history of surgery and pain	27	46.37 ± 1.01	45	48	
	With a history of surgery but not pain	35	41.86 ± 1.09	40	44	

SAS, the self-rating anxiety scale. * represents *P* < 0.01, and the difference is statistically significant.

### Comparison and Correlation Analysis of Preoperative Anxiety and Early Postoperative Pain Between the Two Groups

The SAS score was 40.56 ± 4.72 points in group A and 41.42 ± 4.18 points in group B, and a *t*-test was used to compare SAS scores between the two groups. The difference was not statistically significant. The NRS scores of 50 patients in group A were between 1–5 points, while those of 50 patients in group B were all between 1–7 points. The NRS score of group A was 4.32 ± 0.89 points, and that of group B was 4.90 ± 1.31 points. A *t*-test was used to compare the score between the two groups. The difference was statistically significant (*P* < 0.05). Pearson’s correlation analysis was performed on SAS scores and NRS scores of the two groups. *R* values were 0.836 and 0.8709, respectively. All were greater than 0.5; that is, there was a positive correlation between postoperative pain and preoperative anxiety, and the correlation was significant. Patients with higher preoperative anxiety have stronger postoperative pain (Table 4).

**TABLE 4:** The preoperative anxiety and postoperative pain of the two groups of patients.

Group	SAS	*n*	Mean ± SD	*t*	NRS	*n*	Mean ± SD	*t*-value	*r*
A (*n* = 50)	≤30	0	40.56 ± 4.72	−0.965	≤3	14	4.32 ± 0.89	−2.585	0.836
	31–40	21			4–5	36			
	41–49	29			6–7	0			
	≥50	0			>7	0			
B (*n* = 50)	≤30	0	41.42 ± 4.18		≤3	14	4.90 ± 1.31		0.870
	31–40	20			4–5	10			
	41–49	30			6–7	26			
	≥50	0			>7	0			

*Group A: dexmedetomidine postoperative analgesia pump group.*

*Group B: sufentanil postoperative analgesia pump group.*

*NRS, numerical rating scale; SAS, the self-rating anxiety scale; SD, standard deviation.*

## Discussion

### Preoperative Anxiety

Anxiety is the most common negative emotion and mental disorder in the perioperative period. Disease, hospital environment, preoperative examination and treatment, operations, and postoperative pain may cause anxiety. The results of our survey revealed that the preoperative SAS scores of patients undergoing laparoscopic hysterectomy were significantly higher than the Chinese norm, suggesting that the patient had anxiety before the operation.

In addition, the results of the present study revealed that in these 100 patients, no patient had a SAS score lower than 30, suggesting that anxiety is common in patients. The reason may be related to the fact that the subjects were all women. A related study revealed that female patients were more likely to have anxiety (Mimic et al., 2018). Another reason may be that due to the impact of prevention and control of COVID-19, admission procedures were more complicated than before. The number of family members and friends who visited and took care of the patients was limited. These all aggravated the patients’ anxiety.

The SAS scores of all patients in this study were less than 50. The highest preoperative SAS score was 48, and the patients with SAS scores of 48 accounted for 5% of all patients (Figure 1). This shows that although the patients had preoperative anxiety, the anxiety remained controllable. This may be related to the lower ASA grade of the included patients and the exclusion of patients with malignant tumors and severe complications. In addition, compared with open surgery, laparoscopic surgery has smaller wound sites, less discomfort, faster postoperative recovery, and shorter hospital stays, so patients’ anxiety about postoperative discomfort decreased before their operation.

### Factors Leading to Preoperative Anxiety

There are few studies on the influence of educational levels on preoperative anxiety and postoperative pain. Lanitis revealed that (Lanitis et al., 2015) patients with different education levels have different anxiety levels. Compared with patients with higher education backgrounds, patients with lower educational backgrounds experienced a higher degree of pain (Lanitis et al., 2015). Patients with high educational levels can query the information of related diseases in a variety of ways and can correctly predict their own disease development and prognosis. But this study revealed that the education level of patients had no impact on preoperative anxiety. This may be related to the sample size of this study and the type of operation. Therefore, more research is needed to identify anxiety-prone people accurately to carry out psychological intervention as soon as possible.

This study revealed that the preoperative SAS scores of patients with different occupations were different. The SAS scores of unemployed people, farmers, workers, and personnel of enterprises and institutions were ordered from high to low in turn, and the anxiety level of unemployed people was the highest. The difference in occupation led to the difference in income, and patients with poor financial status were more likely to be anxious.

The patient’s previous surgical experience affected their preoperative anxiety. The SAS scores ranged from high to low in patients with painful operation experience, patients who have had surgery but no painful experience, in patients without operation experience. Patients with previous painful surgery experiences were more prone to anxiety, and the distressing memory of the previous operation aggravated the anxiety.

### Postoperative Pain

Pain is an unpleasant sensation and an emotional experience caused by tissue injury or potential injury (Gómez-de Diego et al., 2014). It is affected by physiological, sensory, emotional, cognitive, sociocultural, and behavioral factors (Melzack and Wall, 1965; Loeser and Melzack, 1999). Pain can be divided into acute pain and chronic pain. Because of the short pain time of laparoscopic surgery (Hartwig et al., 2017), this study was confined to the study of acute postoperative pain.

This survey revealed that the NRS scores of both groups A and B were above 4, and the goal of postoperative pain management was postoperative pain score <3. A total of 72% of patients experienced moderate postoperative pain, suggesting that the acute postoperative pain of patients with laparoscopic hysterectomy has not been effectively controlled.

Acute pain was the most common problem after surgery and has not been effectively controlled (Apfelbaum et al., 2003; Sobol-Kwapinska et al., 2016). A report points out that postoperative pain is not adequately managed in greater than 80% of patients in the United States (Gan, 2017). If pain cannot be fully controlled, acute postoperative pain is harmful and may cause great distress to patients (Schug, 2011; Gravani et al., 2020; Shen et al., 2020).

Traditional concepts believed that laparoscopic surgery has less body surface damage and less postoperative acute pain. Therefore, the postoperative analgesia needs of these patients are often ignored. The results of the study conducted by El Shobary et al. (2006) revealed that although laparoscopic surgery had the advantage of reducing postoperative pain, effective analgesia was still needed to reduce the incidence of complications. The results of this study also suggest this.

### Preoperative Anxiety Is an Important Predictor of Postoperative Pain

The results of this study revealed that preoperative anxiety situations were significantly positively correlated with postoperative pain degrees in patients undergoing laparoscopic hysterectomy. That is, preoperative anxiety is a predictor of postoperative pain. The higher the preoperative anxiety is, the greater the postoperative pain. This is consistent with the results of many previous studies (Suffeda et al., 2016; Mimic et al., 2018). It is worth noting that acute postoperative pain was a predictor of pain after discharge (Kain et al., 2000). The degree of postoperative pain was greatly affected by the degree of anxiety, and the increase of postoperative pain stimulated the anxiety reaction again, forming a cycle of pain and anxiety. Pain can lead to anxiety, and anxiety can enhance pain (Spivey et al., 2018; Gravani et al., 2020; Hah et al., 2020).

### Comparison of Two Postoperative Analgesia Schemes

An important part of breaking the cycle of anxiety is to strengthen postoperative pain management. The results of previous studies revealed that physical therapy, music (Binns-Turner et al., 2011), psychological intervention (Scheel et al., 2014; Hansen et al., 2015), and melatonin could reduce preoperative anxiety and play a role in reducing postoperative pain. However, opioids were still the standard treatment for acute postoperative pain (Gan, 2017).

In this study, dexmedetomidine and opioids were used for postoperative analgesia in patients undergoing laparoscopic hysterectomy. The degree of pain was lower in patients in group A who were treated with dexmedetomidine combined with sufentanil than patients in group B, who were treated with sufentanil alone. In group A, the scores of all patients were below 6, and in group B, the scores of all patients were below 7. This is related to the sedative, analgesic, and anti-anxiety effects of dexmedetomidine (Gu et al., 2020). Chen et al. (2017) used dexmedetomidine in patients undergoing open hysterectomy. The result revealed that dexmedetomidine could reduce postoperative pain. This is consistent with the results of this study. Other studies revealed that the combination of the two drugs could reduce the dosage of opioids and the occurrence of adverse reactions (Almarakbi and Kaki, 2014; Jin et al., 2017; Hamaya et al., 2019), suggesting that the combination of the two drugs is indeed an optimal analgesic scheme. However, ethnicity may be a factor affecting preoperative anxiety and postoperative pain. Conclusions from this research may not be generalizable to other population. Further studies in different ethnic groups are needed.

## Conclusion

In summary, preoperative anxiety is an important predictor of postoperative pain. Patients with higher preoperative anxiety have greater postoperative pain. Patients undergoing laparoscopic hysterectomy have preoperative anxiety, and the degree of anxiety is influenced by the occupation and previous operation experience of the patients. Early and accurate identification of anxiety-prone people before operations will be of great significance to the use of a more optimized analgesia scheme to reduce postoperative pain. Effective postoperative analgesia is conducive to the recovery from disease. We should not neglect the management of postoperative pain because of the small trauma of laparoscopic surgery. The combination of dexmedetomidine and opioids can play an important role in analgesia and improve the quality of anesthesia.

## Data Availability Statement

The original contributions presented in the study are included in the article/supplementary material, further inquiries can be directed to the corresponding author/s.

## Ethics Statement

The studies involving human participants were reviewed and approved by Ethics Committee of Shanxi Provincial People’s Hospital. The patients/participants provided their written informed consent to participate in this study.

## Author Contributions

LZ and L-JH conceived the idea and conceptualized the study. X-LH collected the data. Y-LW, L-SJ, and R-NS analyzed the data. LZ drafted and reviewed the manuscript. All authors read and approved the final draft.

## Conflict of Interest

The authors declare that the research was conducted in the absence of any commercial or financial relationships that could be construed as a potential conflict of interest.

## Publisher’s Note

All claims expressed in this article are solely those of the authors and do not necessarily represent those of their affiliated organizations, or those of the publisher, the editors and the reviewers. Any product that may be evaluated in this article, or claim that may be made by its manufacturer, is not guaranteed or endorsed by the publisher.
